# Gene expression in the corneal endothelium of Fuchs endothelial corneal dystrophy patients with and without expansion of a trinucleotide repeat in TCF4

**DOI:** 10.1371/journal.pone.0200005

**Published:** 2018-07-02

**Authors:** Eric D. Wieben, Ross A. Aleff, Xiaojia Tang, Krishna R. Kalari, Leo J. Maguire, Sanjay V. Patel, Keith H. Baratz, Michael P. Fautsch

**Affiliations:** 1 Department of Biochemistry and Molecular Biology, Mayo Clinic, Rochester, Minnesota, United States of America; 2 Division of Biostatistics and Bioinformatics and Department of Health Sciences Research, Mayo Clinic, Rochester, Minnesota, United States of America; 3 Department of Ophthalmology, Mayo Clinic, Rochester, Minnesota, United States of America; Colorado State University, UNITED STATES

## Abstract

Fuchs Endothelial Corneal Dystrophy (FECD) is a late onset, autosomal dominant eye disease that can lead to loss of vision. Expansion of a CTG trinucleotide repeat in the third intron of the transcription factor 4 (TCF4) gene is highly associated with FECD. However, only about 75% of FECD patients in the northern European population possess an expansion of this repeat. The remaining FECD cases appear to be associated with variants in other genes. To better understand the pathophysiology of this disease, we compared gene expression profiles of corneal endothelium from FECD patients with an expanded trinucleotide repeat (RE+) to those that do not have a repeat expansion (RE-). Comparative analysis of these two cohorts showed widespread RNA mis-splicing in RE+, but not in RE- samples. Quantitatively, we identified 39 genes in which expression was significantly different between RE+ and RE- samples. Examination of the mutation profiles in the RE- samples did not find any mutations in genes previously associated with FECD, but did reveal one sample with a rare variant of laminin subunit gamma 1 (LAMC1) and three samples with rare variants in the gene coding for the mitochondrial protein peripheral-type benzodiazepine receptor-associated protein 1 (TSPOAP1).

## Introduction

Fuchs endothelial corneal dystrophy (FECD) is a common, heritable degeneration of the corneal endothelium with considerable locus heterogeneity. Mutations in 7 genes (AGBL1, COL8A2, LOXHD1, SLC4A11, TCF4, ZEB1, DMPK) have been shown to be either causal or highly associated with the disease [1–9]. A recent large genome-wide association study of FECD added an additional 3 genes to the candidate list (KANK4, LAMC1 and LINC00970/ATP1B1) and re-confirmed the strongest genetic signal for FECD in a predominantly caucasian U.S. population in the transcription factor 4 (TCF4) gene on chromosome 18 [10]. We have previously shown that an expansion of ≥45 CTG trinucleotide repeats in the third intron of TCF4 is highly associated with late onset FECD [9]. This observation has subsequently been replicated in other studies [11, 12] and disease severity has been correlated with the length of repeat expansion [12].

Despite the progress in identifying the genetic underpinnings of FECD, our understanding of the pathogenic pathways in this disease remains limited. In TCF4-associated FECD, the CTG trinucleotide repeat expansion leads to nuclear accumulations of transcribed repeat (CUG)_n_ RNA which sequesters critical RNA splicing factors and leads to widespread splicing changes [13, 14]. Other than TCF4, the myotonic dystrophy-causing CTG repeat in the DM1 protein kinase (DMPK) gene is the only other repeat expansion implicated in FECD [5]. The remaining genes that have been implicated in FECD do not harbor repeat expansions nor have obvious interconnections or shared common pathways. In addition, the previously described loci still fail to explain the entire genetic contribution to FECD, suggesting that additional loci remain to be discovered.

In this study we examined corneal endothelial tissue from eyes with FECD to analyze for differences in RNA splicing patterns and gene expression between subjects with TCF4 trinucleotide repeat expansion (RE+) and those without this expansion (RE-). We also identified several rare genetic variants that may be novel contributors to the genetic basis of FECD.

## Methods

### Isolation of corneal tissue

Patients with FECD (modified Krachmer grade 5 or 6) requiring corneal transplantation and control participants without guttae (grade 0) were enrolled in a Mayo Clinic Institutional Review Board-approved Hereditary Eye Disease Study. Patients that participated in the study provided written informed consent and agreed to a blood draw and use of their excised central corneal endothelium/Descemet membrane specimen obtained at endothelial keratoplasty for FECD. DNA was isolated from peripheral blood leukocytes and RNA was isolated from corneal endothelium/Descemet membrane specimens following storage in RNAlater ICE (ThermoFisher Scientific, Waltham, MA). This research was conducted in accordance with the Declaration of Helsinki.

### RNA isolation and sequencing

A total of 24 corneal endothelial samples were collected in succession and processed for RNASeq at three different times over a 5-year period (2013–2017). Samples in each batch (Table 1) were processed and sequenced in the same manner and sequenced on the same machine approximately 14 months apart, although cDNA synthesis and sequencing methodology varied slightly between the 3 batches (described below). Total RNA was isolated from tissue samples by homogenization in QIAzol Lysis Reagent, chloroform extraction and the RNeasy Mini QIAcube Kit (Qiagen, Valencia, CA) [13, 14]. All samples that had RNA integrity number (RIN) values of ≥6.0 were used in this study. RNA libraries were prepared for each corneal endothelial tissue sample using TruSeq RNA sample Prep kit version 1 or 2 (Illumina, San Diego, CA, USA). Ribosomal transcripts were depleted from total RNA and deoxythymidine triphosphate (dTTP) was replaced with deoxyuridine triphosphate (dUTP) during reverse transcription. DT-priming (Batch #1) or random priming (Batch #2 and #3) were used to generate the second strand synthesis using TruSeq stranded total library preparation kit (Illumina). Due to the differences in priming, Batch #1 was extended in a non-strand specific manner while Batches #2 and #3 were extended in a strand specific manner. The resulting libraries were minimally amplified to enrich for fragments using adapters on both ends and then quantified for sequencing at three samples/lane using a HiSeq2000 (Batch #1) or HiSeq4000 (Batches #2 and #3) Illumina sequencers. Due to differences in cDNA priming, cDNA extension, and sequencing on different machines between batches, each batch was analyzed as its own experimental group to reduce cross variability between batches. Datasets have been uploaded to the Gene Expression Omnibus (GEO) under accession number GSE112201.

**Table 1:** Characteristics of patient samples.

Batch #1 (8 comparisons)				Repeat Length			
RE+			RIN	Short	Long	Gender	Age
2011–024	RNA16	FECD with expansion (RE+)	7.5	12	75	F	56
2011–041	RNA15	FECD with expansion (RE+)	7.8	17	85	M	78
6004	RNA19	FECD with expansion (RE+)	7.3	12	81	M	63
2011–020	RNA10	FECD with expansion (RE+)	7.7	25	56	F	75
**RE-**							
2011–038	RNA20	FECD NO expansion (RE-)	7.6	12	19	F	81
2011–291	RNA79	FECD NO expansion (RE-)	5.9	23	26	F	79
Batch #2 (11 comparisons)							
**RE+**							
2286	RNA90	FECD with expansion (RE+)	6.2	19	71	F	59
2011–359	RNA100	FECD with expansion (RE+)	7.9	12	78	F	78
4827	RNA102	FECD with expansion (RE+)	7.9	12	48	F	80
2011–096	RNA111	FECD with expansion (RE+)	7.1	19	72	M	85
2011–353	RNA112	FECD with expansion (RE+)	7.2	15	84	F	78
2011–313	RNA 120	FECD with expansion (RE+)	6.8	16	80	M	67
2253	RNA27	FECD with expansion (RE+)	7.4	15	77	F	57
2255	RNA28	FECD with expansion (RE+)	7.3	15	79	M	75
2011–392	RNA124	FECD with expansion (RE+)	6.6	32	69	F	69
2011–369	RNA121	FECD with expansion (RE+)	6.1	15	61	M	78
2011–344	RNA114	FECD with expansion (RE+)	6.3	12	60	F	69
**RE-**							
2011–398	RNA87	FECD NO expansion (RE-)	7.1	15	23	M	60
Batch #3 (9 comparisons)							
**RE+**							
2011–553	RNA183	FECD with expansion (RE+)	6.6	79	86	M	77
2011–573	RNA193	FECD with expansion (RE+)	6.2	15	74	F	68
2011–414	RNA141	FECD with expansion (RE+)	6.0	19	91	F	65
**RE-**							
2011–395	RNA142	FECD NO expansion (RE-)	8.6	19	33	F	67
2011–492	RNA166	FECD NO expansion (RE-)	6.5	16	18	F	63
2011–491	RNA167	FECD NO expansion (RE-)	8.0	12	12	F	61

RIN–RNA Integrity Number

### Validation of differential splicing events by RT-PCR

Preparation of cDNA by reverse transcription, amplification by polymerase chain reaction (RT-PCR), and analysis by agarose gel electrophoresis was described previously [13, 14]. Briefly, Platinum PCR Super Mix High Fidelity (Invitrogen, Carlsbad, CA) was used to amplify approximately 40 ng of genomic DNA isolated from FECD patients corneal endothelium following transplantation. PCR conditions for ADD3 and INF2 amplification were as follow: 95 °C for 6 minutes (1 cycle), 95 °C denaturation for 1 minute, 58 °C annealing for 1 minute, and 68 °C extension for 4 min (40 cycles), and a 7 minute 68 °C extension. Similar conditions were performed for CADM1 except that the annealing temperature was 66 °C. Specific primers for the amplification are provided in Table 2.

**Table 2:** Primer sequences for RT-PCR.

Primers	Sequence
**ADD3**	
5-ADD3	5’-CAGGACCACAATCTCAGTTGC-3’
3-ADD3	5’-TCGCTTAGCAAGCTCATCTTC-3’
**CADM1**	
5-CADM1	5’-GCCTGTGATGGTAACTTGGGTG-3’
3-CADM1	5’-CCCCAGAATGATGAGCAAGCAC-3’
**INF2**	
5-Inf2	5’-GAAGCGAAGGAAGAAGCGT-3’
3-Inf2	5’-TTTAGGAAGCAGGTGGGAGG-3’
**TCF4 trinucleotide repeat length**	
5-TCF-Fuchs	5’- CAGATGAGTTTGGTGTAAGATG-3’
3-TCF-Fuchs1	5’-ACAAGCAGAAAGGGGGCTGCAA-3’
5-FAM-TCF-Fuchs	5’-CAGATGAGTTTGGTGTAAGATG-3’

### Analysis of differentially spliced genes

Whole transcriptomic sequencing data were analyzed using a comprehensive computational pipeline (MAP-RSeq) [15] to align, assess and deliver multiple genomic features. MAP-RSeq uses a variety of freely available bioinformatics tools along with in-house developed methods to obtain in-depth quality control data, transcriptome read alignment, abundance of gene expression, exon expression, and other transcriptomic features. The Binary Alignment Map (BAM) files from MAP-RSeq were then analyzed using MISO (Mixture of Isoforms) [16] packages that quantify the expression level of alternatively spliced genes between groups. For pairwise comparisons, MISO calculates Bayesian probabilities and calculates a percent spliced in (PSI) for every skipped-exon event (range from 0 to 1, with 0 being completely excluded and 1 being uniformly included in the splicing products) which identifies genuine differences in the splicing of a given exon between two samples. To identify differentially spliced genes, we utilized stringent filtering criteria (reads to support inclusive isoform is >2; reads to support exclusive isoform is >2; sum of inclusive and exclusive reads is at least 25; PSI change >0.2, Bayes factor >50) within MISO to perform genome-wide pairwise comparisons between RE+ and RE- FECD samples within each batch of samples (28 total comparisons). Due to differences in sample preparation and sequencing instruments, like prepared samples were batched together and compared to each other. This was to reduce potential variability within the analysis. The comparisons for batches 1 and 3 were performed by R package edgeR (https://academic.oup.com/bioinformatics/article/26/1/139/182458). For batch 2, we analyzed the data using a z-test using the assumption that the biological variation of gene expression in the FECD no expansion sample was similar to that of the FECD with expansion group. Once all comparisons within a batch were performed, results were compared between the 3 batches. Genes that were identified in 2 of the 3 batch comparisons and in 12 or more comparisons were reported.

### DNA isolation and trinucleotide repeat characterization

TCF4 trinucleotide repeat length was determined as described previously [13, 14]. Briefly, leukocyte-derived DNA was extracted using AutoGen FlexiGene (Qiagen) and suspended in 1x TE for a final concentration of 250 ng/μl. Trinucleotide repeat regions from each sample were PCR amplified in an iCycler (Bio-Rad, Hercules, CA.) by placing 100 ng of genomic DNA with 10 pmoles of oligonucleotide primers specific for TCF4 (5-TCF-Fuchs and 3-TCF-Fuchs1, Table 2) in the presence of Invitrogen Platinum PCR Super Mix High Fidelity. The PCR program used for amplification was as follows: Hot Start 95 °C for 6 min. (1 cycle); 95 °C denaturation for 1 min., 62 °C annealing for 1 min., 68 °C extension for 3 min. (35 cycles); 68 °C for 7 min. (1 cycle); and followed by a 4 °C hold.

For Short Tandem Repeat analysis, a 5’ FAM primer (5-FAM-TCF-Fuchs, Table 2) was used in place of 5-TCF-Fuchs and PCR was performed as described above. After PCR amplification, 2 μl of DNA was mixed with 12 μl of diluted Map Marker 1000 Bio Ventures Inc. (Murfreesboro, TN.). Gene Scan was carried out using ABI 3730XL DNA Analyzer (Foster City, CA.).

### Pathway analysis using PANTHER

Gene sets identified by filtering of MISO results were analyzed for overrepresentation of genes in specific PANTHER families and pathways using the default settings of the PANTHER web portal, including Bonferroni correction for multiple testing (http://www.pantherdb.org/).

## Results

### Sample characteristics

The twenty-four corneal endothelium samples used for this study are described in Table 1. All samples were obtained during corneal endothelial replacement surgery for FECD. Eighteen samples were from RE+ patients (mean age = 71 yrs.; range = 56–85 yrs.; CTG repeat length ≥45) and six were from RE- patients (mean age = 68 yrs.; range = 60–81 yrs.; CTG repeat length <45). Five of 6 patients (83%) in the RE- group were female, whereas 11/18 (61%) patients in the RE+ group were female. Samples were collected over a 5-year period and grouped into 3 batches based on like RNA preparation and RNA sequencing techniques (Table 1). RNA sequencing was performed for each sample within each batch. Pairwise comparisons for gene splicing and expression between RE+ and RE- samples were performed within each batch, which allowed for 8, 11 and 9 comparisons in batches 1, 2 and 3 respectively, for a total of 28 pairwise comparisons.

### Alternative splicing

To evaluate mRNA splicing differences between RE+ and RE- samples, we used MISO software as a screening tool. Splicing events that were common among all 3 batches that met our stringent cutoff criteria resulted in 20 differential splicing events (Table 3). Notably, splicing events in MBNL1, NUMA1 and PPFIBP1 were found in all 28 pairwise comparisons, whereas mis-splicing in INF2, SCARB1, SYNE1, ADD3 and MBNL2 was identified in >90% of the comparisons.

**Table 3:** Differential splicing events.

Gene Name	Event name	Sum Comparisons (28 total)	RE+FECD (percent spliced in)	RE-FECD (percent spliced in)	RE-Control (percent spliced in)
**RE+ vs RE- splicing differences**					
**MBNL1**	chr3:152163071:152163328:+@chr3:152164493:152164546:+@chr3:152165409:152165562:+	28	0.86	0.40	0.39
**NUMA1**	chr11:71723941:71727306:-@chr11:71723447:71723488:-@chr11:71721832:71721900:-	28	0.27	0.73	0.77
**PPFIBP1**	chr12:27829361:27829532:+@chr12:27829997:27830029:+@chr12:27832422:27832572:+	28	0.12	0.73	0.64
**INF2**	chr14:105180540:105181193:+@chr14:105181621:105181677:+@chr14:105185132:105185947:+	27	0.2	0.83	0.90
**SCARB1**	chr12:125270903:125271049:-@chr12:125267229:125267357:-@chr12:125262174:125263132:-	27	0.27	0.76	0.75
**SYNE1**	chr6:152469180:152469513:-@chr6:152466622:152466690:-@chr6:152464758:152464900:-	27	0.26	0.77	0.75
**ADD3**	chr10:111890121:111890244:+@chr10:111892063:111892158:+@chr10:111893084:111895323:+	26	0.39	0.05	0.05
**MBNL2**	chr13:97999058:97999321:+@chr13:98009050:98009103:+@chr13:98009736:98009889:+	26	0.60	0.03	0.06
TTC7A	chr2:47183978:47184146:+@chr2:47185634:47185691:+@chr2:47202112:47202242:+	25	0.06	0.35	0.23
ARVCF	chr22:19959409:19959494:-@chr22:19958739:19958858:-@chr22:19957402:19958266:-	24	0.82	0.4	0.38
**TSPOAP1**	chr17:56387328:56387519:-@chr17:56385902:56386741:-@chr17:56385203:56385302:-	24	0.24	0.93	0.93
NDUFV3	chr21:44317037:44317157:+@chr21:44323292:44324386:+@chr21:44328974:44329773:+	24	0.31	0.66	0.56
APBB2	chr4:40946882:40947087:-@chr4:40937094:40937156:-@chr4:40936631:40936716:-	23	0.43	0.80	0.71
IFI44	chr1:79124997:79125168:+@chr1:79128389:79128563:+@chr1:79129450:79129763:+	23	0.61	0.90	0.82
**EXOC1**	chr4:56749989:56750094:+@chr4:56755054:56755098:+@chr4:56756389:56756552:+	22	0.81	0.39	0.16
**ITGA6**	chr2:173362703:173362828:+@chr2:173366500:173366629:+@chr2:173368819:173371181:+	22	0.18	0.68	0.69
**CLASP1**	chr2:122204913:122205083:-@chr2:122203025:122203072:-@chr2:122187649:122187753:-	22	0.21	0.82	0.87
**COPZ2**	chr17:46105838:46105876:-@chr17:46105042:46105155:-@chr17:46103533:46103841:-	21	0.35	0.05	0.10
**CD46**	chr1:207958964:207959027:+@chr1:207963598:207963690:+@chr1:207966864:207968861:+	20	0.30	0.54	0.60
CADM1	chr11:115080294:115080377:-@chr11:115069126:115069158:-@chr11:115049364:115049495:-	18	0.26	0.03	0.03
**Found in FECD RE+ vs controls but not in RE+ vs RE-**					
FGFR1	chr8:38314874:38315052:-@chr8:38287200:38287466:-@chr8:38285864:38285953:-	23	0.84	0.33	0.35
VEGFA	chr6:43746626:43746655:+@chr6:43749693:43749824:+@chr6:43752278:43754223:+	19	0.78	0.58	0.5
AKAP13	chr15:86198648:86199018:+@chr15:86201768:86201821:+@chr15:86207794:86207986:+	19	0.53	0.82	0.76
GOLGA2	chr9:131036129:131036251:-@chr9:131035064:131035144:-@chr9:131030699:131030803:-	18	0.09	0.35	0.44
NHSL1	chr6:138768138:138768330:-@chr6:138763120:138763251:-@chr6:138751530:138754817:-	18	0.80	0.46	0.37
KIF13A	chr6:17794480:17794626:-@chr6:17790103:17790141:-@chr6:17788007:17788106:-	17	0.12	0.63	0.73
PLEKHM2	chr1:16046229:16046415:+@chr1:16047824:16047883:+@chr1:16051812:16052040:+	17	0.29	0.69	0.65
MYO6	chr6:76618213:76618344:+@chr6:76621389:76621415:+@chr6:76623780:76623998:+	15	0.11	0.32	0.41
ABI1	chr10:27065994:27066170:-@chr10:27060004:27060018:-@chr10:27059174:27059274:-	13	0.38	0.83	0.8
KIF13A	chr6:17772139:17772290:-@chr6:17771345:17771449:-@chr6:17763924:17765177:-	12	0.47	0.19	0.18

**Fig 1:**
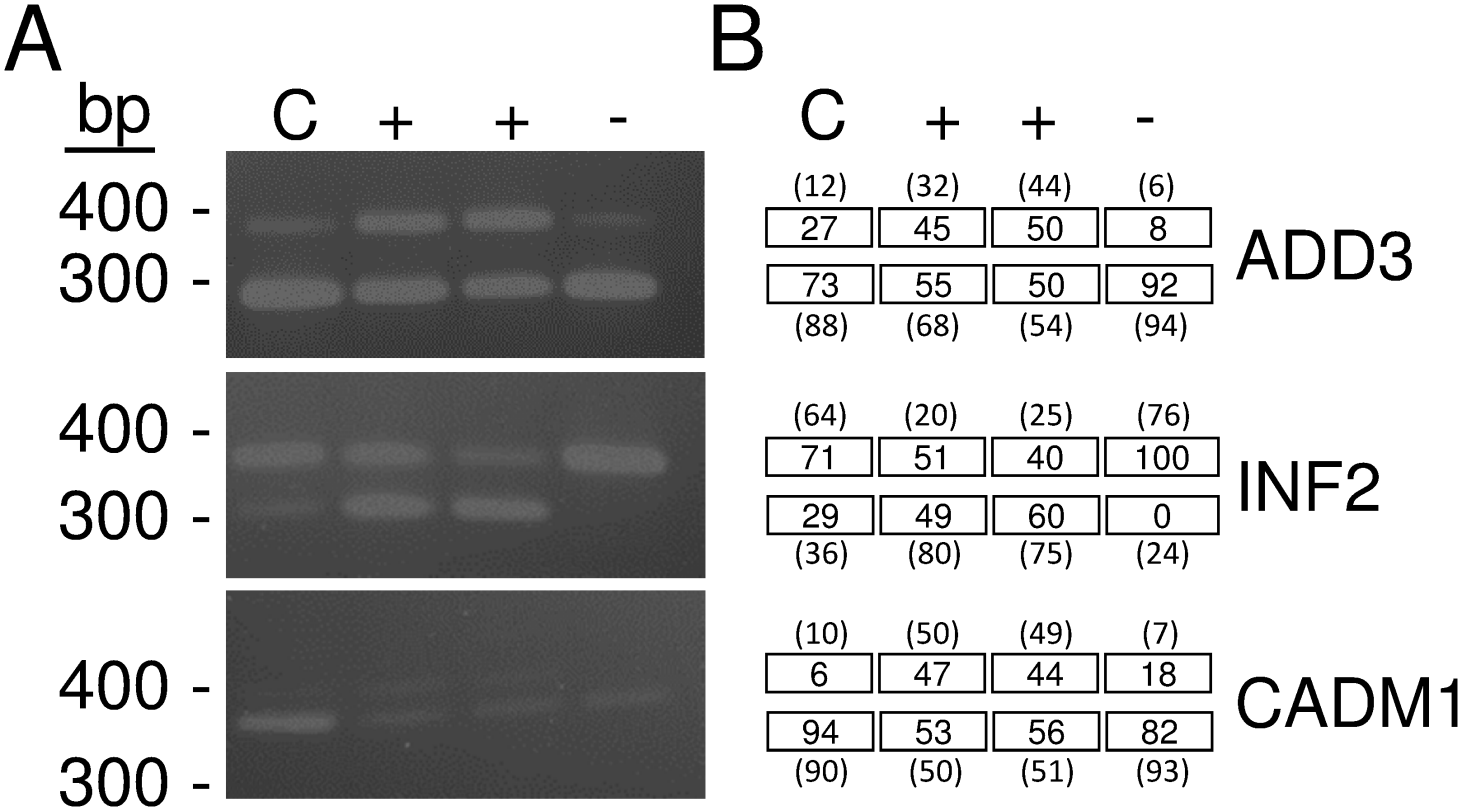
Validation of RNA Sequencing identified mis-splicing events. (A) RT-PCR amplified products derived from specific primers (Table 2) that flanked selected exons in the ADD3, INF2, and CADM1 genes. Amplification of a larger DNA fragment in ADD3 and CADM1 (exon inclusion) and a smaller DNA fragment in INF2 (exon exclusion) are shown in samples obtained from two independent FECD patients that have a TCF4 trinucleotide repeat expansion (denoted with a plus sign). In contrast, these bands are either lacking or in reduced amounts in a sample from a FECD patient that does not contain a TCF4 trinucleotide repeat expansion (denoted with a minus sign) or a non-FECD patient sample (labeled with a C). (B) Numbers in boxes represent percentage of PCR products containing inclusion/exclusion of exons for each sample in (A). Numbers in parentheses are percent spliced in values obtained from RNASeq on the same samples from which PCR was performed. These results confirm exon inclusion and exclusion as identified by RNA sequence and PCR analysis.

To assess several of the mis-splicing events identified by MISO analysis, we performed RT-PCR on FECD samples obtained from RE+ and RE- patients (Fig 1). In the ADD3, INF2, and CADM1 gene, the RE- sample (shown as the minus sign in Fig 1) showed amplification of a fragment that corresponded to the same size as the non-FECD sample (labeled as C in Fig 1). In contrast, the two RE+ samples (shown with a plus sign in Fig 1) showed 2 bands of similar intensity, one representing the same size as the control/RE- samples, and a larger band in the ADD3 and CADM1 genes representing the inclusion of an additional exon sequence in each gene. In INF2, a smaller band was identified in the RE+ samples suggesting the exclusion of exon sequence within this gene. Densitometry provided percentage of inclusion/exclusion for each band (Fig 1B, values in boxes), which correlated with percent spliced in values obtained by RNASeq (Fig 1B, value in parentheses). Additional PCR validation of MBNL1, MBNL2, NUMA1, SYNE1, PPFIBP1, ITGA6 and CLASP1 has previously been reported in the context of RE+ vs. non-FECD control tissue, which are similar events identified in this study [13,14].

### Differential gene expression

Quantitative differences in gene expression between RE+ and RE- samples were identified for each of the 3 batches of samples. Genes with a minimum log2 fold change of 1 were further compared between batches. Comparison of genes with a minimum log2 fold change of 1 between the 3 gene sets identified 28 genes in which expression was increased in RE+ compared to RE- samples and 11 genes in which expression was decreased (Table 4). Overrepresentation analysis of the 39 genes using Panther did not reveal any significant gene ontology term enrichments.

**Table 4:** Differential gene expression between RE+ and RE- corneal endothelial sample.

Gene	logFC-1	p value-1	FDR-1	logFC-2	p value-2	FDR-2	logFC-3	p value-3	FDR-3	Function
**RE+ up**										
AGR3	-2.96	8.92E-04	2.61E-02	-6.41	1.81E-17	5.62E-15	-2.05	1.50E-05	1.28E-03	disulfide isomerase
AIFM3	-4.26	9.89E-06	9.87E-04	-1.85	5.86E-08	3.53E-06	-1.17	2.09E-03	6.02E-02	apoptosis inducer, mitochondrial
ANO9	-3.76	2.14E-04	9.75E-03	-4.70	5.00E-11	6.07E-09	-2.19	2.06E-03	5.96E-02	calcium activated chloride channel
AP1M2	-4.88	1.24E-08	3.89E-06	-2.49	1.98E-04	3.94E-03	-1.82	1.59E-06	1.95E-04	protein sorting in golgi
CDO1	-2.14	2.18E-03	4.32E-02	-2.02	3.71E-14	7.61E-12	-2.47	3.49E-08	8.47E-06	cysteine dioxygenase
COCH	-3.26	6.03E-05	4.10E-03	-3.23	8.89E-05	2.05E-03	-3.13	1.72E-09	8.52E-07	collagen binding
CRISP2	-2.97	7.36E-04	2.28E-02	-6.30	1.13E-03	1.59E-02	-2.20	5.04E-03	0.11	cysteine rich secretory protein
CSPG5	-2.77	4.80E-04	1.67E-02	-3.31	8.42E-06	2.85E-04	-1.08	2.67E-02	0.34	proteoglycan
DLL3	-3.56	2.82E-03	4.91E-02	-3.73	2.06E-04	4.05E-03	-1.15	4.29E-03	0.10	Notch ligand
ENOX1	-3.42	6.69E-06	7.40E-04	-2.95	1.08E-03	1.54E-02	-1.87	1.85E-05	1.49E-03	hydroquinone (NADH) oxidase activity
EYA4	-2.95	1.92E-03	3.98E-02	-5.58	5.97E-09	4.79E-07	-5.74	1.50E-13	2.55E-10	protein phosphatase
FRZB	-5.88	1.65E-10	8.00E-08	-4.65	2.96E-04	5.41E-03	-3.20	1.58E-06	1.95E-04	modulator of Wnt signalling
GALNT8	-4.58	2.07E-06	3.05E-04	-4.60	2.39E-06	9.83E-05	-3.78	9.17E-13	9.36E-10	N-acetylgalactosaminyltransferase
HPGD	-5.31	3.22E-08	9.17E-06	-4.46	2.44E-06	1.00E-04	-3.82	1.10E-21	8.45E-18	prostaglandin metabolism
KRTAP5-10	-1.95	2.09E-02	0.13	-3.66	3.65E-03	3.85E-02	-1.01	8.81E-03	0.17	keratin associated protein
LRRN4CL	-1.56	2.36E-02	0.14	-3.44	2.92E-06	1.16E-04	-1.08	2.13E-02	0.30	membrane protein
MAFA	-2.17	1.55E-02	0.12	-5.79	3.60E-18	1.24E-15	-5.19	3.03E-13	3.86E-10	transcription factor
MAL2	-1.72	1.65E-02	0.12	-2.39	8.68E-05	2.02E-03	-1.10	1.47E-02	0.23	transcytosis protein
MAP7D2	-1.65	4.12E-02	0.18	-2.37	3.82E-07	1.89E-05	-1.10	1.32E-02	0.21	cytoskeletal protein
NPM2	-2.87	1.18E-04	6.58E-03	-1.71	5.33E-04	8.77E-03	-1.77	2.18E-03	6.19E-02	chromatin reprogramming
NRG3	-5.29	3.76E-07	7.41E-05	-5.26	3.38E-16	9.68E-14	-2.09	2.31E-04	1.10E-02	receptor tyrosine kinase binding
NTF4	-2.92	7.75E-04	2.38E-02	-1.81	1.69E-04	3.47E-03	-1.42	2.93E-04	1.35E-02	neurotrophic factor
PRSS8	-4.53	3.68E-05	2.82E-03	-5.23	2.41E-04	4.59E-03	-1.53	1.15E-03	3.92E-02	serine protease
ROBO2	-1.91	4.87E-02	0.20	-2.42	5.03E-03	4.84E-02	-2.59	3.91E-05	2.75E-03	transmembrane receptor
SFRP4*	-3.3	2.18E-03	4.31E-02	-3.96	8.45E-04	1.26E-02	-1.03	0.18	0.91	modulator of Wnt signalling
SLC10A4	-2.62	1.72E-03	3.77E-02	-2.81	5.73E-04	9.25E-03	-3.72	7.65E-13	8.37E-10	bile acid:sodium symporter
SLC5A1	-6.07	2.35E-06	3.38E-04	-2.41	2.32E-03	2.76E-02	-3.27	7.76E-15	2.38E-11	sodium-glucose cotransporter
VSIG2	-1.9	6.50E-03	7.63E-02	-4.06	6.43E-07	3.01E-05	-1.16	1.76E-03	5.33E-02	plasma membrane protein
**RE+ down**										
BICC1	3.1	1.13E-03	2.97E-02	2.37	4.01E-05	1.08E-03	1.27	5.01E-02	0.50	negative regulator of Wnt signalling
CASZ1	1.81	1.47E-02	0.11	1.73	1.96E-05	6.02E-04	1.86	1.65E-04	8.43E-03	transcription factor
CDKN2C	1.74	1.13E-02	0.10	3.07	2.98E-08	1.98E-06	2.13	1.20E-06	1.58E-04	cell growth regulation
DLEU1	2.6	1.99E-03	4.04E-02	2.68	2.28E-04	4.40E-03	1.55	4.25E-03	0.10	Long ncRNA
EDIL3	7.62	1.97E-15	2.42E-12	7.04	3.97E-06	1.50E-04	2.32	1.05E-02	0.19	integrin ligand
F2RL2	2.88	4.92E-05	3.47E-03	3.62	3.64E-03	3.84E-02	1.72	4.35E-03	0.10	G protein coupled receptor
GALNT14	2.84	8.97E-04	2.62E-02	2.36	4.60E-03	4.54E-02	1.30	9.32E-03	0.17	N-acetylgalactosaminyltransferase
GPRC5B	3.24	7.29E-06	7.76E-04	2.79	2.91E-03	3.25E-02	2.08	7.85E-04	0.03	G protein coupled receptor
MYLK	2.41	2.27E-03	4.43E-02	2.82	2.83E-04	5.22E-03	1.12	3.25E-02	0.38	myosin light chain kinase
SERPINA3	2.04	1.71E-03	3.76E-02	3.06	4.07E-04	7.04E-03	2.08	2.11E-05	1.66E-03	protease inhibitor
SYNPO2	1.76	2.59E-02	0.15	1.79	1.71E-03	2.21E-02	1.14	9.20E-03	0.17	actin binding

* Statistical significance not obtained in batch 3 comparison; FDR–False Discovery Rate

### Characterization of novel genetic variants

**Fig 2:**
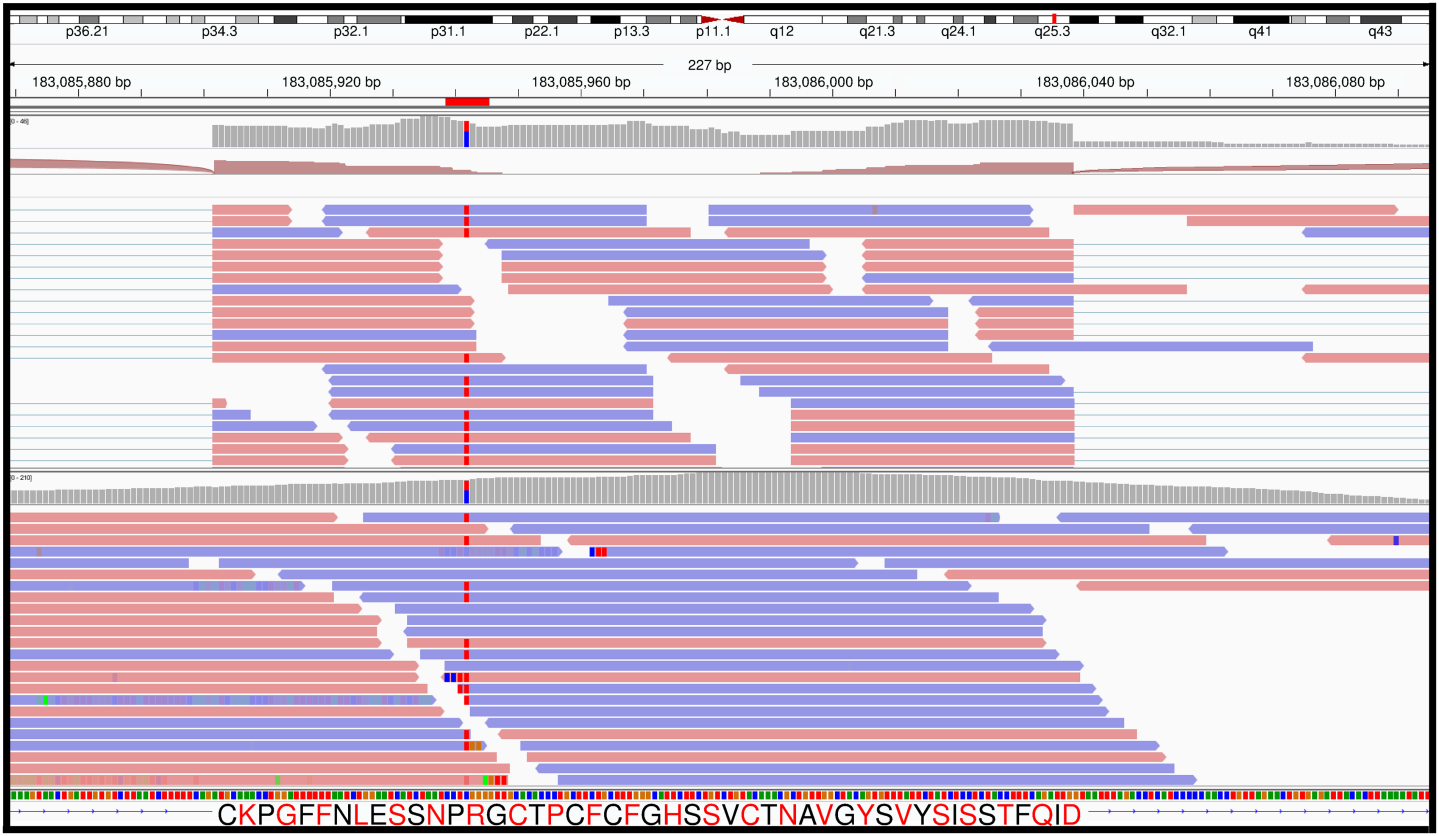
Identification of a rare LAMC1 variant in RNA79 (from patient 2011–291). RNASeq (top panel) and exome sequencing data (bottom panel) from the same individual are shown. The location of a C->T variant which leads to the R490W substitution in the LAMC1 protein is highlighted in both panels. Images were obtained from the Integrative Genomics Viewer.

To further analyze the RE- samples, we assessed whether these samples contained mutations in any of the other FECD associated genes. Using RNASeq data, ZEB1, SLC4A11, KANK4, ATP1B1 and COL8A2 were expressed in RE- cells, but no known FECD variants or other rare mutations were identified in these genes. AGBL1 and LOXHD1 were not expressed in any of our corneal endothelium (RE- or RE+) samples, so we were unable to evaluate possible variants in these genes from RNASeq data. Exome sequencing of leukocyte DNA from each of the RE- patients confirmed these observations. We did identify a rare mutation in LAMC1 in one of the RE- samples. Sample RNA79 had a heterozygous C->T variant at chr1: 183085942, leading to an arginine to tryptophan substitution at amino acid 490 (R490W) (Fig 2). This variant was detected in both the RNASeq data and in leukocyte DNA exome sequencing of this patient. Analysis of this C->T variant in the Exome Aggregation Consortium (ExAC) browser, which utilizes data obtained from numerous independent investigators performing large scale sequencing projects, showed that it was only observed 61 times in 121,388 analyzed chromosomes suggesting a rare nucleotide change (allele frequency = 5.0 x 10^−4^).

**Fig 3:**
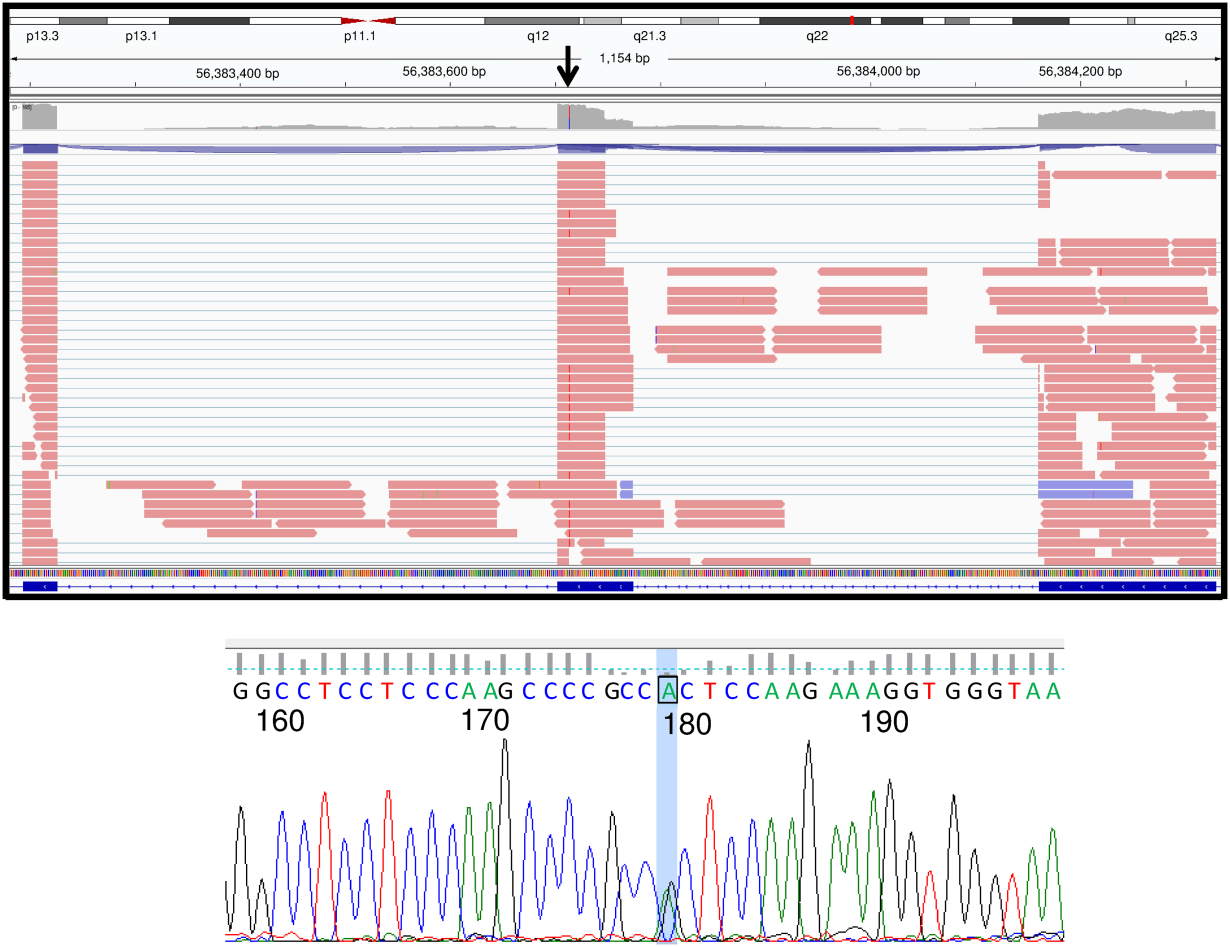
Identification of a rare TSPOAP1 variant in RNA142 (from patient 2011–395). RNASeq data (top panel) and a Sanger sequencing trace from patient 2011–395 DNA (bottom panel) are shown. Top panel image obtained from the Integrative Genomics Viewer.

**Fig 4:**
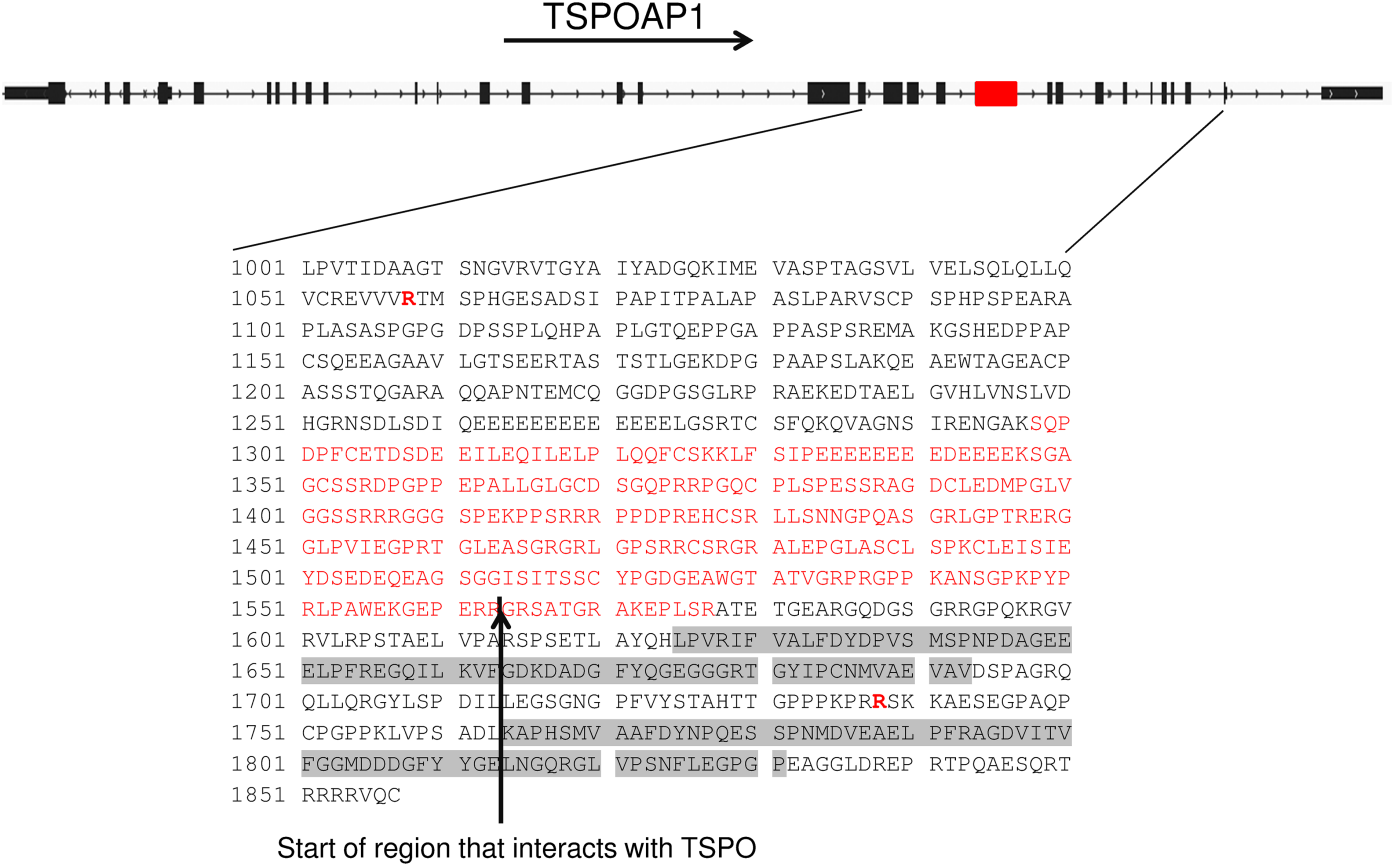
TSPOAP1 variants in FECD patients. The locations of two substitution variants in the primary sequence of TSPOAP1 are shown in bold red (positions 1058 and 1738) under a diagram of the structure of the TSPOAP1 gene. The exon that is preferentially excluded from RE+ samples by alternative splicing is shown in red, and the location of this sequence in the TSPOAP1 protein is also shown in red (position 1298–1577). The vertical black arrow designates the start of the region of the TSPOAP1 protein that is thought to interact with TSPO.

We also assessed genetic variation in RE- samples of the mis-spliced genes in RE+ samples (Table 3). We identified a rare hg19 chr17:g.56383714 C>T variant, resulting in an arginine to histidine substitution at amino acid 1738 (R1738H), in the TSPOAP1 (formerly designated BZRAP1) gene in RE- sample RNA142 (Fig 3). This variant has been found only once in the ExAC browser, leading to a calculated allele frequency of only 4.7 x 10^−5^. Sanger sequencing of leukocyte DNA from this patient confirmed the presence of this variant (Fig 3B). In RE+ samples, mis-splicing of TSPOAP1 led to the deletion of 280 amino acids, which removes part of the motif that has been shown to interact with TSPO, a mitochondrial outer membrane protein involved in the transport of cholesterol into mitochondria (Fig 4).

**Fig 5:**
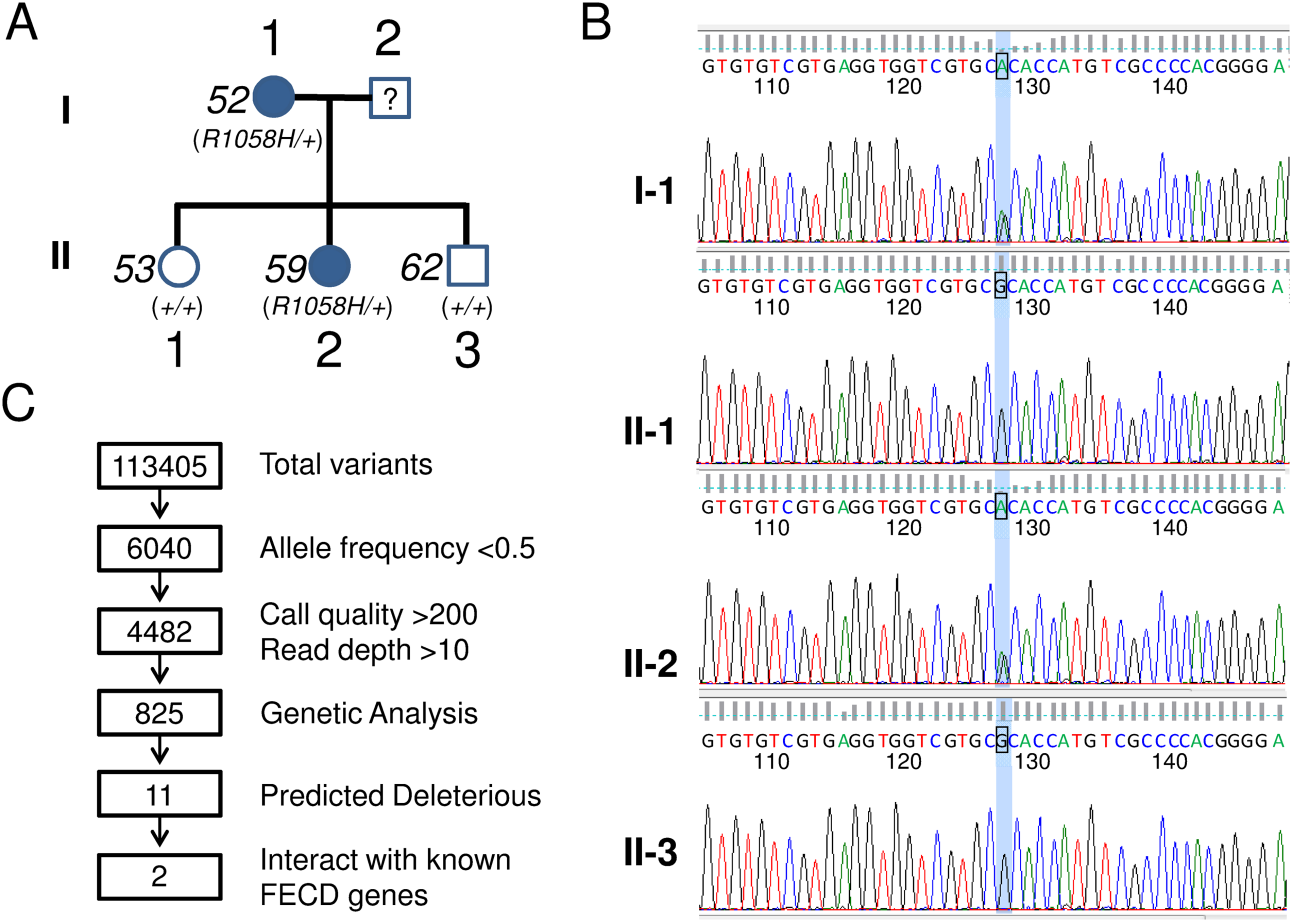
Identification of TSPOAP1 variants in a family with RE- FECD. (A) Pedigree of RE- FECD family. Patient 52 (91 years old) and patient 59 (64 year old) had modified Krachmer scores of 6 in both eyes and TCF4 trinucleotide repeat sizes of 18, 24 and 24, 31 respectively. Patient 53 (66 years old) and patient 62 (52 years old) had modified Krachmer scores of 0 in both eyes and TCF4 trinucleotide repeat sizes of 24, 31 and 18, 32 respectively. Outside of the FECD diagnosis, there were no evident medical conditions or syndromic diseases that were common within the pedigree other than solitary skin cancers in 2 of the 4 family members. (B) Sanger sequencing traces of DNA from the vicinity of the R1058H variant are shown. Both affected family members (I-1 and II-2) are confirmed to be heterozygous for the R1058H variant. (C) Schematic diagram showing the filtering strategy used to identify variants in exome sequencing of 4 family members. The number of variants remaining after each filtering step is shown in the boxes.

The finding of a rare TSPOAP1 variant in one of our RE- RNASeq samples led us to further investigate this genes sequence in affected members of a RE- FECD family we had previously identified. In this family, we identified and validated a different rare TSPOAP1 variant in two affected members within this family (Fig 5). As shown in the pedigree in Fig 5A, an hg19 chr17:g.56388483 C->T variant results in an arginine to histidine substitution at amino acid 1058 (R1058H). This particular variant was found only 5 times in 58,162 chromosomes analyzed in the ExAC database (allele frequency 8.6 x 10^−5^). This particular DNA sequence variation was not found in the two unaffected siblings of the affected daughter in this family. These variants were identified in exome sequencing studies and have been confirmed by Sanger sequencing (Fig 5B).

Within this family, the TSPOAP1 variant was one of eleven rare variants that co-segregated with FECD (Fig 5C). Of these eleven candidates, only two (PLEKHF1 and TSPOAP1) have known interactions with well-established FECD genes. The Y64* variant identified in PLEKHF1 [allele frequency = 5.0 x 10^−5^ in ExAC database (6/121152)] also segregated with disease in this family and would clearly influence the function of the affected allele. Additionally, the PLEKHF1 gene is known to bind with ZEB1, a well-characterized FECD gene. However, PLEKHF1 is expressed at very low levels in our RNASeq data from the corneal endothelium.

## Discussion

FECD is a debilitating eye disease with genetic association to multiple genes. Of these genes, an expansion of a CTG repeat sequence in the TCF4 gene is by far the most common genetic anomaly found in FECD patients in the United States [1, 11, 12]. In the current study, we found that mRNA splicing patterns in RE- corneal endothelium samples differ from those in RE+ corneal endothelium. This reinforces the premise that the mis-splicing events seen in the RE+ samples are a direct consequence of the expansion of the CTG repeats at the TCF4 locus rather than a secondary response to the disease process. In addition to these qualitative observations among 18 RE+ samples and 6 RE- samples, we also identified quantitative differences in gene expression between RE+ and RE- samples. Splicing patterns in RE- samples generally mirror those of control endothelium. Of the 20 differential splicing events found in comparisons of RE+ and RE- samples (Table 3), all but one (IFI44) were identified in a previous study comparing FECD RE+ samples to controls [14]. Interestingly, 14 of these 20 events were found in the group of top 24 differentially spliced genes between controls and RE+ samples (Table 3, genes in bold font). In the same study, we compared alternative splicing in RE+ FECD to the well-characterized mis-splicing due to a similar CTG repeat expansion in the untranslated region of the DMPK gene in myotonic dystrophy, type 1. The list of top mis-splicing events is strikingly similar between FECD and DM1, and the finding of nuclear foci containing expanded (CUG)_n_ and sequestered muscleblind protein further suggests a shared pathogenic mechanism.

The 10 alternative splicing events identified in previous comparisons [14] between RE+ samples and controls that did not meet criteria in the RE+ to RE- comparisons (Table 3, bottom) are also of potential interest. In principle, these are alternative splicing events that differ between controls and FECD, regardless of the TCF4 expansion status. A closer examination of these 10 splicing events revealed that all but 2 actually met our stringent criteria in two of the three sequencing batches and were detected in approximately half of the pairwise comparisons (Table 3). The 2 splicing events that distinguished both RE+ and RE- FECD from controls were in transcripts from ABI1 and KIF13A, but even these 2 genes were noted in over 40% of the pairwise RE+/RE- comparisons. Therefore, we cannot conclude that any splicing event distinguishes FECD, regardless of expansion status, from control tissue or that any mis-splicing event is common to all variants of this genetically heterogeneous disease.

Reproducible quantitative changes in gene expression between RE+ and RE- corneal endothelium samples were identified for a set of 39 genes. These represent a set of genes that are differentially regulated by the repeat expansion in TCF4, but changes in the expression of these genes do not appear to be critical for the development of FECD. These data demonstrate that expansion of the CTG repeat in TCF4 leads to widespread changes in gene expression. While this dataset did not reveal any common pathway involvement, several genes can be found in the same pathway. For example, FRZB and SFRP4 are two modulators of Wnt signaling that are upregulated in RE+ samples. Further analysis of the complete dataset may provide novel insights into their molecular and physiological associations.

The identification of rare variants in TSPOAP1, a gene exhibiting mis-splicing in both FECD and DM1, in three RE- patients from two separate families is also noteworthy. TSPOAP1 is a TSPO-binding protein, and it has also been shown to bind to TCF4. TSPO is thought to be important in the regulation of mitophagy, which is thought to be important in the pathogenesis of FECD [17–19]. Although the functional consequences of TSPOAP1 binding of TSPO are unknown, it can be speculated that structural variants of TSPOAP1 might have a differential effect on the activity of TSPO, which might in turn contribute to FECD pathogenesis.

Although the family data also revealed alternative variants that could be related to the pathogenesis of FECD, the segregation of the TSPOAP1 variants with disease in our family data is intriguing and suggests possibilities for common mechanisms between RE+ samples and at least a subset of RE- samples. Specifically, RE+ patients also produce significant amounts of a variant TSPOAP1 mRNA that codes for 280 fewer amino acids compared to the predominant form found in the corneal endothelium from RE- and control patients. Thus, the majority of all FECD patients in our population are likely to be producing a variant TSPOAP1 isoform as a result of the splicing changes that result from expansion of the CTG repeat in TCF4.

Clearly the RE- group of FECD patients is heterogeneous, because there is considerable locus heterogeneity in the 20–25% of FECD patients in our population that are not RE+. Within the RE- group characterized by RNASeq studies here, we did not identify any samples with rare variants in well-established FECD genes (SLC4A11, COL8A2, ZEB1, TCF4) but we did find that one patient had a rare variant in LAMC1, which has been implicated as an FECD gene by a recent large GWAS effort [10].

Of note, we did not identify expression of LOXHD1 or AGBL1 in any of our samples, whether RE+ or RE-. These genes have been implicated in FECD in prior studies, but this lack of expression in corneal endothelial tissue in adults brings into question whether genetic variants in these genes could have a functional role in the development of the disease.

The limitations of this study include limited statistical power due to the analysis of small groups of samples and the heterogeneity of the RE- group. Additionally, due to consecutive sample collection and processing, batch #2 only had 1 RE- sample to perform analysis with. While this analysis appears limited, it is strengthened by the fact that we took data obtained from each batch and cross compared them to each other and only reported genes that were identified in 2 out of 3 comparisons and in 12 or more independent comparisons. This analysis plan would have eliminated most if not all of the false positives obtained within batch 2 due to a single RE- comparison. While the findings of differences in splice patterns and gene expression are likely to be significant, we cannot ensure that pathologically important differences were not missed. Resolving this limitation will depend not only on larger sample sizes but also in the further elucidation of the true causative genetic variants in the RE- group. Given the genetic mutations and implicated genes described in prior literature, the absence of those variants in this current RE- study group, and our finding of two novel variants, RE- FECD may very well represent a phenotype resulting from a very diverse source of genetic variants and pathways.

The work presented here supports our previous conclusion that corneal endothelium from RE+ patients exhibit characteristic mRNA splicing events, and we now confirm that many of these abnormalities are not present in the corneal endothelium from RE- patients. These qualitative differences in gene expression are supplemented by quantitative differences in the expression of at least 39 genes. These findings suggest that there are real biological differences between RE+ FECD and RE- FECD corneas and lend support to a hypothesis that mis-splicing is a key pathogenic feature of RE+ FECD but not RE- FECD. Therefore, future efforts to identify genetic influences on the development of FECD should consider stratification of study populations according to repeat status. This should also apply to studies of the basic science of the disease, such as cell culture systems in which RE+ or RE- models may express differential biology and differential responses to interventions or therapeutics. In addition, we identified a novel candidate locus, TSPOAP1, and confirm that a rare variant in a recently described locus, LAMC1, was found in a subset of RE- FECD.

## References

[1] Baratz KH, Tosakulwong N, Ryu E, Brown WL, Branham K, Chen W, et al E2-2 protein and Fuchs’s corneal dystrophy. N Engl J Med. 2010;363:1016–24. doi: 10.1056/NEJMoa1007064 20825314 10.1056/NEJMoa1007064

[2] Gattey D, Zhu AY, Stagner A, Terry MA, Jun AS. Fuchs endothelial corneal dystrophy in patients with myotonic dystrophy: a case series. Cornea. 2014;33:96–8. doi: 10.1097/ICO.0000000000000018 24270677 10.1097/ICO.0000000000000018 PMC3898337

[3] Gottsch JD, Sundin OH, Liu SH, Jun AS, Broman KW, Stark WJ, et al Inheritance of a novel COL8A2 mutation defines a distinct early-onset subtype of fuchs corneal dystrophy. Invest Ophthalmol Vis Sci. 2005;46:1934–1939. doi: 10.1167/iovs.04-0937 15914606 10.1167/iovs.04-0937

[4] Kuot A, Hewitt AW, Griggs K, Klebe S, Mills R, Jhanji V, et al Association of TCF4 and CLU polymorphisms with Fuchs’ endothelial dystrophy and implication of CLU and TGFBI proteins in the disease process. Eur J Hum Genet. 2012;20:632–638. doi: 10.1038/ejhg.2011.248 22234156 10.1038/ejhg.2011.248 PMC3355250

[5] Mootha VV, Hansen B, Rong Z, Mammen PP, Zhou Z, Xing C, et al Fuchs’ Endothelial Corneal Dystrophy and RNA Foci in Patients With Myotonic Dystrophy. Invest Ophthalmol Vis Sci. 2017;58:4579–4585. doi: 10.1167/iovs.17-22350 28886202 10.1167/iovs.17-22350 PMC5590687

[6] Riazuddin SA, Parker DS, McGlumphy EJ, Oh EC, Iliff BW, Schmedt T, et al Mutations in LOXHD1, a recessive-deafness locus, cause dominant late-onset Fuchs corneal dystrophy. Am J Hum Genet. 2012;90:533–539. doi: 10.1016/j.ajhg.2012.01.013 22341973 10.1016/j.ajhg.2012.01.013 PMC3309196

[7] Riazuddin SA, Vithana EN, Seet LF, Liu Y, Al-Saif A, Koh LW, et al Missense mutations in the sodium borate cotransporter SLC4A11 cause late-onset Fuchs corneal dystrophy. Hum Mutat. 2010;31:1261–1268. doi: 10.1002/humu.21356 20848555 10.1002/humu.21356 PMC2970683

[8] Riazuddin SA, Zaghloul NA, Al-Saif A, Davey L, Diplas BH, Meadows DN, et al Missense mutations in TCF8 cause late-onset Fuchs corneal dystrophy and interact with FCD4 on chromosome 9p. Am J Hum Genet. 2010;86:45–53. doi: 10.1016/j.ajhg.2009.12.001 20036349 10.1016/j.ajhg.2009.12.001 PMC2801746

[9] Wieben ED, Aleff RA, Tosakulwong N, Butz ML, Highsmith WE, Edwards AO, et al A common trinucleotide repeat expansion within the transcription factor 4 (TCF4, E2-2) gene predicts Fuchs corneal dystrophy. PLoS One. 2012;7:e49083 doi: 10.1371/journal.pone.0049083 23185296 10.1371/journal.pone.0049083 PMC3504061

[10] Afshari NA, Igo RPJ, Morris NJ, Stambolian D, Sharma S, Pulagam VL, et al Genome-wide association study identifies three novel loci in Fuchs endothelial corneal dystrophy. Nat Comm. 2017;8:14898. 10.1038/ncomms14898 PMC5379100 28358029

[11] Mootha VV, Gong X, Ku HC, Xing C. Association and familial segregation of CTG18.1 trinucleotide repeat expansion of TCF4 gene in Fuchs’ endothelial corneal dystrophy. Invest Ophthalmol Vis Sci. 2014;55:33–42. doi: 10.1167/iovs.13-12611 24255041 10.1167/iovs.13-12611 PMC3880006

[12] Vasanth S, Eghrari AO, Gapsis BC, Wang J, Haller NF, Stark WJ, et al Expansion of CTG18.1 Trinucleotide Repeat in TCF4 Is a Potent Driver of Fuchs’ Corneal Dystrophy. Invest Ophthalmol Vis Sci. 2015;56:4531–4536. doi: 10.1167/iovs.14-16122 26200491 10.1167/iovs.14-16122 PMC4515948

[13] Du J, Aleff RA, Soragni E, Kalari K, Nie J, Tang X, et al RNA toxicity and missplicing in the common eye disease fuchs endothelial corneal dystrophy. J Biol Chem. 2015;290:5979–5990. doi: 10.1074/jbc.M114.621607 25593321 10.1074/jbc.M114.621607 PMC4358235

[14] Wieben ED, Aleff RA, Tang X, Butz ML, Kalari KR, Highsmith EW, et al Trinucleotide Repeat Expansion in the Transcription Factor 4 (TCF4) Gene Leads to Widespread mRNA Splicing Changes in Fuchs’ Endothelial Corneal Dystrophy. Invest Ophthalmol Vis Sci. 2017;58:343–352. doi: 10.1167/iovs.16-20900 28118661 10.1167/iovs.16-20900 PMC5270622

[15] Kalari KR, Nair AA, Bhavsar JD, O’Brien DR, Davila JI, Bockol MA, et al MAP-RSeq: Mayo Analysis Pipeline for RNA sequencing. BMC Bioinformatics. 2014;15:224 doi: 10.1186/1471-2105-15-224 24972667 10.1186/1471-2105-15-224 PMC4228501

[16] Katz Y, Wang ET, Airoldi EM, Burge CB. Analysis and design of RNA sequencing experiments for identifying isoform regulation. Nat Methods. 2010;7:1009–1015. doi: 10.1038/nmeth.1528 21057496 10.1038/nmeth.1528 PMC3037023

[17] Benischke AS, Vasanth S, Miyai T, Katikireddy KR, White T, Chen Y, et al Activation of mitophagy leads to decline in Mfn2 and loss of mitochondrial mass in Fuchs endothelial corneal dystrophy. Sci Rep. 2017;7:6656 doi: 10.1038/s41598-017-06523-2 28751712 10.1038/s41598-017-06523-2 PMC5532298

[18] Gatliff J, East D, Crosby J, Abeti R, Harvey R, Craigen W, et al TSPO interacts with VDAC1 and triggers a ROS-mediated inhibition of mitochondrial quality control. Autophagy. 2014;10:2279–2296. doi: 10.4161/15548627.2014.991665 25470454 10.4161/15548627.2014.991665 PMC4502750

[19] Selvaraj V, Stocco DM. The changing landscape in translocator protein (TSPO) function. Trends Endo Met. 2015;26:341–348. 10.1016/j.tem.2015.02.007 PMC7171652 25801473

